# Surgical treatment of atrial fibrillation in elderly patients undergoing high risk cardiac surgery

**DOI:** 10.1186/s13019-024-02796-7

**Published:** 2024-07-03

**Authors:** Mohammed Mohsin Uzzaman, Imthiaz Manoly, Mohini Pannikkar, Vincenzo Caruso, Maciej Matuszewski, Nicolas Nikolaidis, Stephen Billing

**Affiliations:** Department Of Cardiothoracic Surgery, New Cross Hospital, Wolverhampton, UK

**Keywords:** Atrial fibrillation, Cox-Maze IV, Ablation, Elderly, Outcomes, Long-term

## Abstract

**Background:**

Evaluating outcomes of concurrent Cox-Maze procedures in elderly patients undergoing high-risk cardiac surgery.

**Mehods:**

We retrospectively identified patients aged over 70 years with Atrial Fibrillation (AF) from 2011 to 2017 who had two or more other cardiac procedures. They were subdivided into two groups: 1. Cox-Maze IV AF ablation. 2. No-Surgical AF treatment. A propensity match score was used to generate a homogeneous cohort and to eliminate confounding variables. Heart rhythm was assessed from Holter reports or 12-lead ECG. Follow-up data was collected through telephone consultations and medical records.

**Results:**

There were 239 patients. Median follow up was 61 months. 70 patients had Cox-Maze IV procedures (29.3%). Demographic, intra- and post-operative outcomes were similar between groups although duration of pre-operative AF was shorter in Cox-Maze group (*p* = 0.001). There was no significant 30-day mortality difference in propensity matched cohorts (*n* = 84. *P* = 0.078). Sinus rhythm at annual and latest follow-up was 84.9% and 80.0% respectively in Maze group – 160 patients (66.9%) were alive at long-term follow-up with good survival outcomes in Cox Maze group. There was a high proportion of patients in NYHA 1 status in Cox-Maze group. No differences observed in freedom from stroke (*p* = 0.80) or permanent pacemaker (*p* = 0.33) between the groups.

**Conclusions:**

Surgical ablation is beneficial in elderly patients undergoing high-risk surgery - promoting excellent long-term freedom from AF and symptomatic / prognostic benefits, without added risk. Therefore, surgical risk should not be reason to deny benefits of concomitant AF-ablation.

**Clinical trial registration:**

Not required.

## Background

Atrial fibrillation (AF) is the most common cardiac arrhythmia, with a prevalence of 2.1% in people aged more than 65 years [1] and prevalence is expected to double by 2050 [2]. 50% of patients undergoing mitral valve surgery present with AF [3], as do 1–6% of patients undergoing coronary artery bypass grafting or aortic valve surgery [4]. Several large studies, including the Framingham study, have shown that AF is associated with increased risk for mortality and morbidity [5, 6]. In the past decade, studies have suggested that cardiac surgery patients with AF have reduced survival over time if AF is left untreated [4, 7], and that patients who present with AF have worse perioperative outcomes [8–10].

The Cox maze procedure was originally designed in 1987 as a concomitant procedure for the treatment of AF in patients undergoing MVS [11]. After several iterations, the technically easier and faster Cox-Maze IV procedure was introduced in 2002 [12]. Despite the proven success of the Cox-Maze procedure, referring physicians and cardiac surgeons remain somewhat reluctant to adopt the procedure for surgical ablation of AF. Gammie and colleagues published a study based on the Society of Thoracic Surgeons’ database, which demonstrated that only 38**%** of patients presenting for cardiac surgery while experiencing AF underwent any type of corrective surgical ablation concomitantly with a valve or coronary bypass surgery [13]. The surgical complexity and perceived operative risk are major variables in the decision of whether to perform surgical ablation for AF at the time of other cardiac procedures. Currently, no risk models are available for concomitant arrhythmia surgery; thus, the extent of the additional associated risk has been poorly defined. In addition the level of training required to perform surgical ablation and a lack of recognition of the clinical importance of AF may also contribute to the relatively low uptake of the procedure in clinical practice.

The treatment of elderly higher risk patients with AF remains a challenge due to concurrent morbidities and age-related physiological changes. Anticoagulation to prevent the thromboembolic events associated with AF also has a greater risk of major bleeding complications in elderly patients. Although more elderly patients are undergoing cardiac surgery in the last 15 years [14], few studies have examined the efficacy of surgical AF ablation in this group. This study was to evaluate the outcomes of concomitant Cox maze procedures in elderly patients (aged **≥** 70 years) who undergo higher risk cardiac surgery. We hypothesized that a concomitant Cox maze procedure would not increase the peri-operative risk in such patients.

## Methods

This was a single-center cohort study in which all data were collected retrospectively for surgery occurring between January 2011 and December 2017. We defined the study population as patients with pre-operative AF, who were above 70 years and underwent 2 cardiac procedures with or without additional AF procedures. Patients were divided into two groups based on how the AF was addressed: (1) Cox-maze IV procedure (2) Nil surgical AF treatment group. Patients undergoing redo procedures or who had isolated Pulmonary vein isolation (PVI) or left atrial appendage occlusion (LAAO) to address their AF were excluded. History of preoperative atrial fibrillation was determined through our local database and type of atrial fibrillation was determined according to Heart Rhythm Society guidelines. The database was also used to gain additional preoperative characteristics and perioperative outcomes. Detailed follow-up data was collected for patients through telephone consultations and medical record review. In addition, reports from primary care physicians and cardiologists from referring centers were obtained if required. Rhythm status for patients who underwent a surgical ablation procedure was determined according to the Heart Rhythm Society guidelines and verified by electrocardiogram and Holter monitor. The Heart Rhythm Society definition of success (i.e., all documented atrial Arrhythmias **>** 30 s are considered a failure) was used to determine the return to sinus rhythm rate at first follow-up (usually 6 weeks), annual follow-up and long-term follow-up [15]. Most patients (> 90%) had either a 48–72 h holter monitor at 6 week and 12 month interval. Anticoagulation status was also collected at the follow-up time points. Operative mortality was defined as death occurring within 30 days of operation or at any time point during the index hospitalization.

### Operative approach

Multiple surgeons performed the complete Cox-maze IV lesion set in a standard fashion as described previously [1]. This consisted of a bilateral PVI, roof and floor connecting lesions between the right and left pulmonary veins, lesion to the left atrial appendage, mitral isthmus lesion, right intercaval lesion, right appendage lesion, right free wall lesion to the tricuspid annulus lesion and the coronary sinus lesion. The energy source used was cryothermia and bipolar radiofrequency (Medtronic, Minneapolis, Minn; AtriCure Inc, West Chester, Ohio). The left atrial appendage was occluded in all patients who had Cox-Maze IV. This was performed using the Atriclip device (AtriCure Inc, West Chester, Ohio). The patients in the “Nil procedure” group only had two cardiac procedures and served as our primary control group. The decision of whether to add the Cox Maze procedure to a specific surgical procedure was left to the discretion of the surgeon.

### Statistical analysis

Continuous data are presented as mean +/- standard deviation or Median +/- Interquartile range. Categorical data is presented as frequency (+/-percent) unless otherwise noted. Patient groups were compared using **c**2 or Fisher exact test for preoperative and postoperative categorical variables and student independent samples t test or Mann-Whitney U test for continuous measures as appropriate based on parametric test assumptions. Kaplan-Meier analysis was used to assess the Cox-Maze group on cumulative survival, freedom from AF, NYHA 1 status, freedom from permanent pacemaker insertion and freedom from stroke.

This is a retrospective study that has inherent selection bias. Propensity score matching was therefore performed to create two groups with no difference with respect to confounding factors. Propensity score was estimated using the logistic regression model with AF-Ablation treatment as the primary outcome. The following explanatory variables were included in the analysis: gender, age, Logistic Euroscore, renal function, grade of pulmonary hypertension, concomitant ischemic coronary artery disease, left ventricular ejection fraction and type of AF (chronic, paroxysmal or new onset). Patients were matched using the ‘nearest neighbor’ procedure with a ratio of 1:1 and caliper width of 0.2. Intra- and post-operative variables of the matched group were then compared as above. For all the tests, a *p* value < 0.05, was considered statistically significant. All analyses were conducted using SPSS version 28.0 (SPSS Inc, Chicago, Ill) or GraphPad Prism, Version 6.00 for Mac (GraphPad Software, La Jolla, CA, USA).

## Results

### Patient details

**Fig. 1:**
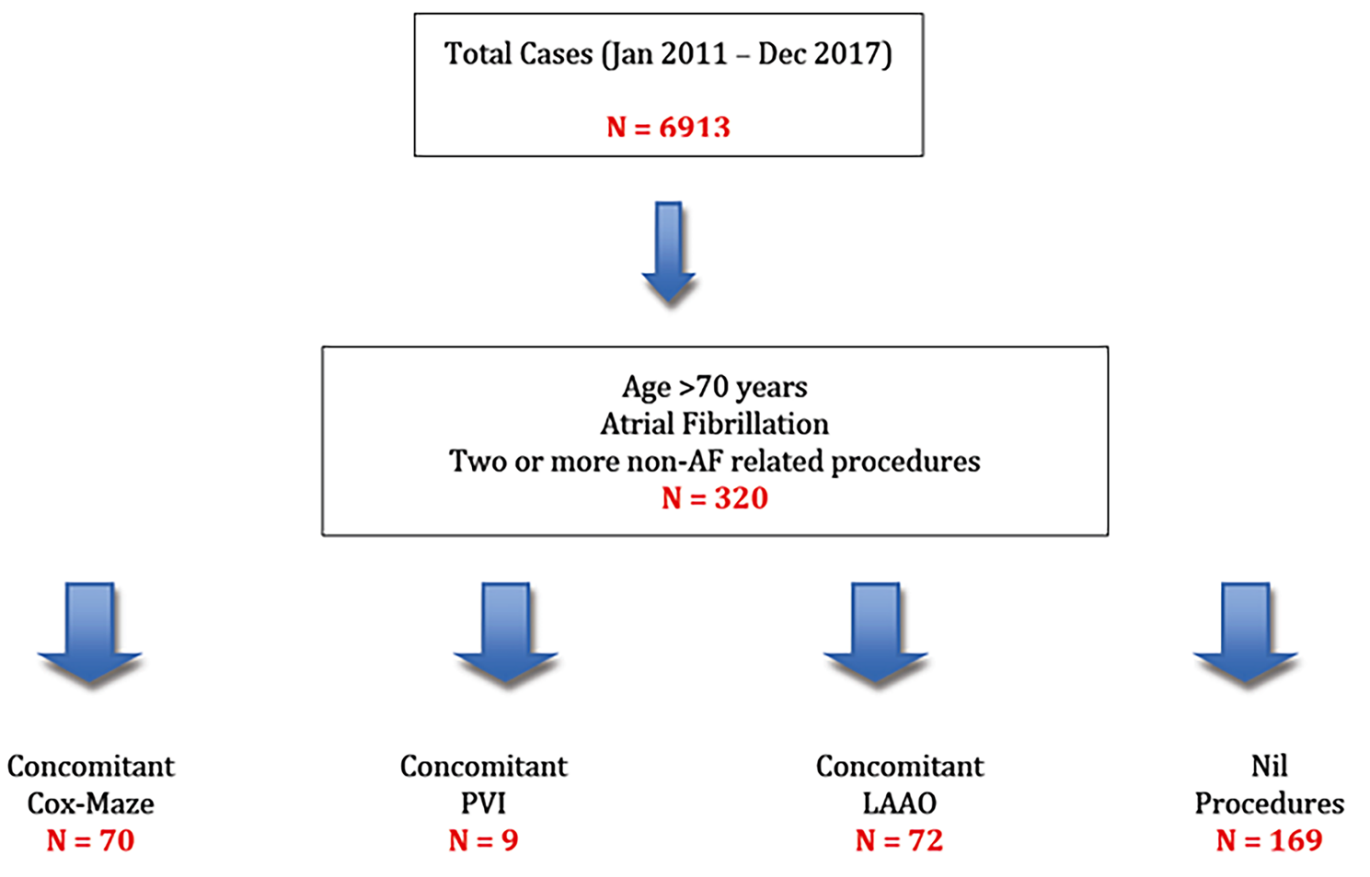
Flow chart of cases performed at our trust

Between Jan 2011 and Dec 2017, we performed 6913 cases at our institution (see Fig. 1). There were 239 patients who were aged over 70 years with pre-operative AF who underwent two cardiac procedures (with or without any additional AF procedures). 70 patients (29.2%) had Cox-Maze IV and 169 patients (70.8%) had No Surgical-AF treatment (Fig. 1). The propensity score matching created a database of 84 patients, matched with a ratio of 1:1 (Cox Maze group, *n* = 42, No Surgical AF treatment *n* = 42). The pre-operative characteristics of both cohorts are summarized in Table 1.

**Table 1:** Demographic data for the two study groups

	Before Matching			After Matching	
	Cox-Maze (*n* = 70)	Nil (*n* = 169)	*p*-value	Cox-Maze (*n* = 42)	Nil (*n* = 42)
**AGE**	76.1+/- 3.7	77.2+/-4.3	0.26	76.2 ± 3	76.4 ± 3
**GENDER**	M 33 F 37	M 89 F 80	0.78	M 17 F 22	M 25 F 20
**DIABETES**	8	39	0.13	4	5
**Creatinine Clearance**	59.3+/-20.0	61.6+/-21.2	0.51	57.6 ± 22.3	63.4 ± 19.9
**Recent MI**	4	6	0.11	-	-
**COPD**	14	27	0.74	-	-
**Body Mass Index**	26.4+/-5.0	27.8+/-4.7	0.22	-	-
**Smoking History**	23	85	0.48	-	-
**Hypertension**	41	106	0.31	-	-
**Previous CVA/TIA**	7	23	0.79	-	-
**NYHA 1**	2	7	0.10	1	0
**NYHA IV**	18	47	0.74	7	8
**Pulmonary HTN**	3	26	0.02	2	0
**Euroscore**	10+/-8.6	13+/-8.9	0.04	9.8 ± 7.5	10.4 ± 6.9
**LA Size**	4.8+/-1.4	5.1+/-1.1	0.48	5 ± 1	5.3 ± 1
**AF Duration**	19.9+/-22.3	94.2+/-113.7	0.001	60 ± 22	62 ± 23
**Paroxysmal AF (%)**	16 (22.9)	12 (7.1%)	0.00001	0(0)	0(0)

The mean age of the whole cohort before matching was 76.6+/-4.1 years. Before matching, the logistic Euroscore was slightly lower in the Cox Maze group (10.0 +/-8.6) compared to the No Surgical AF treatment (13.0+/-8.9) (*p* = 0.04). There were no differences in the NYHA 1 status (*p* = 0.10) or NYHA 4 status (*p* = 0.74) between the groups. There were a higher number of patients with pulmonary hypertension in the No Surgical AF treatment group (15.4%) compared to the AF ablation group (*p* = 0.02). Echocardiogram findings were comparable between the groups with respects to left atrial size (*p* = 0.48) and left ventricular function (*p* = 0.11). There was a significantly shorter duration of AF in the Cox-Maze group (19.9+/-22.3 months) compared to the No Surgical AF treatment group (94.2+/-113.7 months) (*p* = 0.001). There were a significantly higher proportion of patients with paroxysmal AF (PAF) in the Cox-Maze group (22.9%) compared to No Surgical AF treatment groups (*p* = 0.00001).

### Intra-operative factors

The specific intra-operative factors are summarized in Table 2. 194 cases (81.1%) were performed in an elective setting, with no difference between the groups (*p* = 0.48). Unsurprisingly, the majority of patients in the cohort had Mitral valve surgery (186 patients, 77.8%). There were 105 patients (43.9%) who had concomitant CABG with a lower number in the Cox-Maze group (27.1%, *p* = 0.003). Other procedures not listed in Table 2 include aortic valve surgery, tricuspid valve surgery and aortic surgery. The operative times were not significantly higher in the Cox-Maze groups, which may reflect a higher inclination to perform a maze procedure by faster surgeons. Nine patients required IABP at the end of the case (3.8%) with no difference between the groups (*p* = 0.67).

**Table 2:** Intra-operative parameters in the two groups

	Before Matching			After Matching		
	Cox-Maze (*n* = 70)	Nil (*n* = 169)	*P*-VALUE	Cox-Maze (*n* = 42)	Nil (*n* = 42)	*P*-VALUE
XCT (min)	135.2 ± 40.6	130.7 ± 86.3	0.91	140.86 ± 45	126.5 ± 39	0.420
CPB (min)	165.7 ± 64.9	160.4 ± 58.9	0.77	172.57 ± 78	160.8 ± 53	0.514
CABG	19	86	**0.001**	12	17	0.165
Mitral Surgery	51	135	0.23	30	25	0.178

### Perioperative outcomes

Before matching, the overall peri-operative mortality was 6.3% (*n* = 15) with an apparent difference observed between the two groups (Cox-Maze: *n* = 1, 1.7%; Nil Surgical AF treatment: *n* = 14, 8.2%, *p* = 0.047). There were three cases of stroke in the entire cohort (1.2%) which were observed only in the Nil surgical AF treatment group (*p* = 0.21). After matching, there was no peri-operative mortality difference (*n* = 0 vs. 2, *p* = 0.078). There was no difference in the stroke rate in the matched groups either (*p* = 0.080). There were 10 patients (4.2%) who required in hospital-PPM with 3 cases (4.3%) in the Cox-Maze group (*p* = 0.96). The duration of ITU stay (*p* = 0.25) and overall hospital stay (*p* = 0.30) was comparable between the groups. There was no difference in return to theatre (*p* = 0.42) between the groups. Other post-operative outcomes are summarized in Table 3.

**Table 3:** Peri-operative outcomes between the groups

	Before Matching			After Matching		
	Cox-Maze (*n* = 70)	Nil (*n* = 169)	*P*-VALUE	Cox-Maze (*n* = 42)	Nil (*n* = 42)	*P*-VALUE
MORTALITY 30 days	1	14	**0.047**	0	3	0.08
STROKE	0	3	0.21	0	3	0.08
Hospital Stay days (Median + IQR)	11 (8–17)	8 (7–16)	0.30	16.5 (9–18)	12 (8–17)	0.29
Renal failure	6	26	0.16	5	8	0.33
PPM	3	7	0.96	2	3	0.65
Respiratory Complication	18	28	0.06	19	12	0.11
GI complications	2	5	0.50	1	1	0.76
Return to theatre	7	22	0.42	4	8	0.08
ICU Stay (Median)	3 (2–6)	3 (2–7)	0.25	3 (2–5)	3 (2–6)	0.72

### Long-term outcomes

**Fig. 2:**
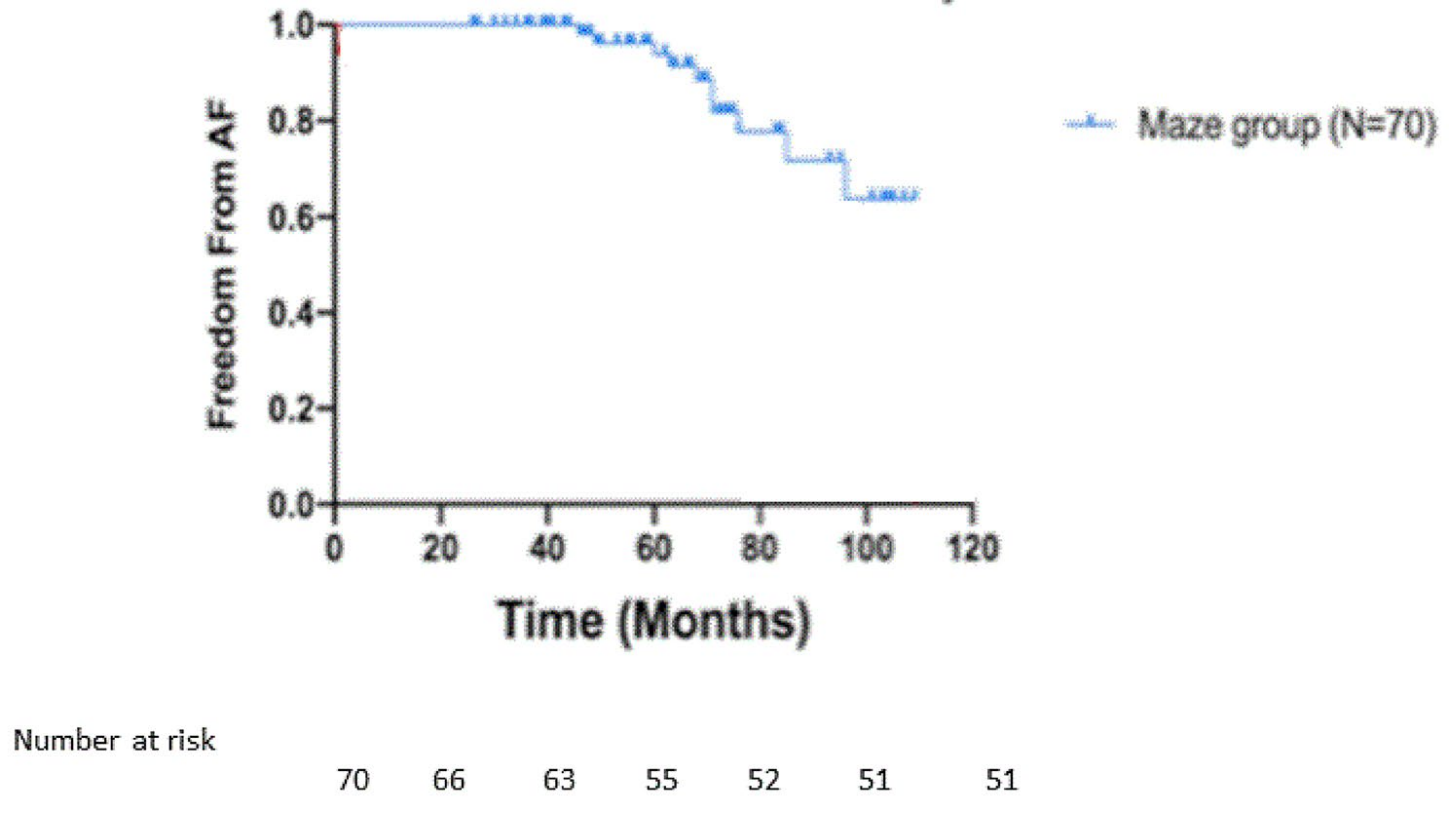
Freedom from AF in Cox-Maze group

The mean follow-up was 58.9 +/-26.4 months (Median 61 months). 17 patients were lost to follow-up giving 92.6% long-term data completion. Freedom from AF in the cox maze group is shown in Fig. 2. At 12 months 84.9% of the Cox Maze group were in SR. These benefits persisted until latest follow-up (80%) at 59+/-20.0 months.

**Fig. 3:**
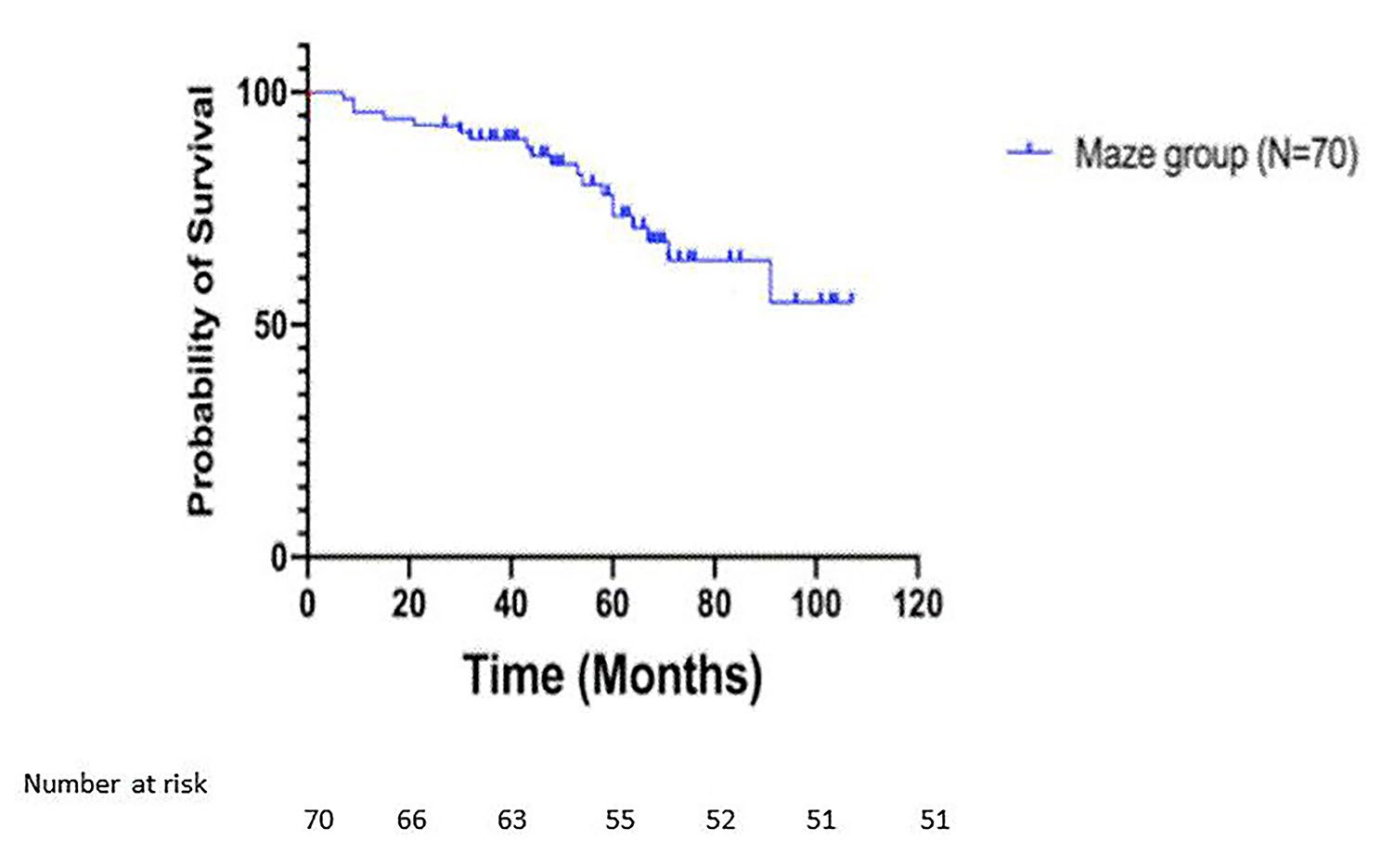
Survival curve in Cox-Maze group

There were 79 mortalities (33.1%) during the follow-up period. The number of patients in the propensity matched groups were too small to perform a meaningful comparison. Figure 3 shows the Kaplain-Meir analysis for the Cox-Maze group. There were 78.6% survival in the Cox-Maze group after 5 years follow-up.

**Fig. 4:**
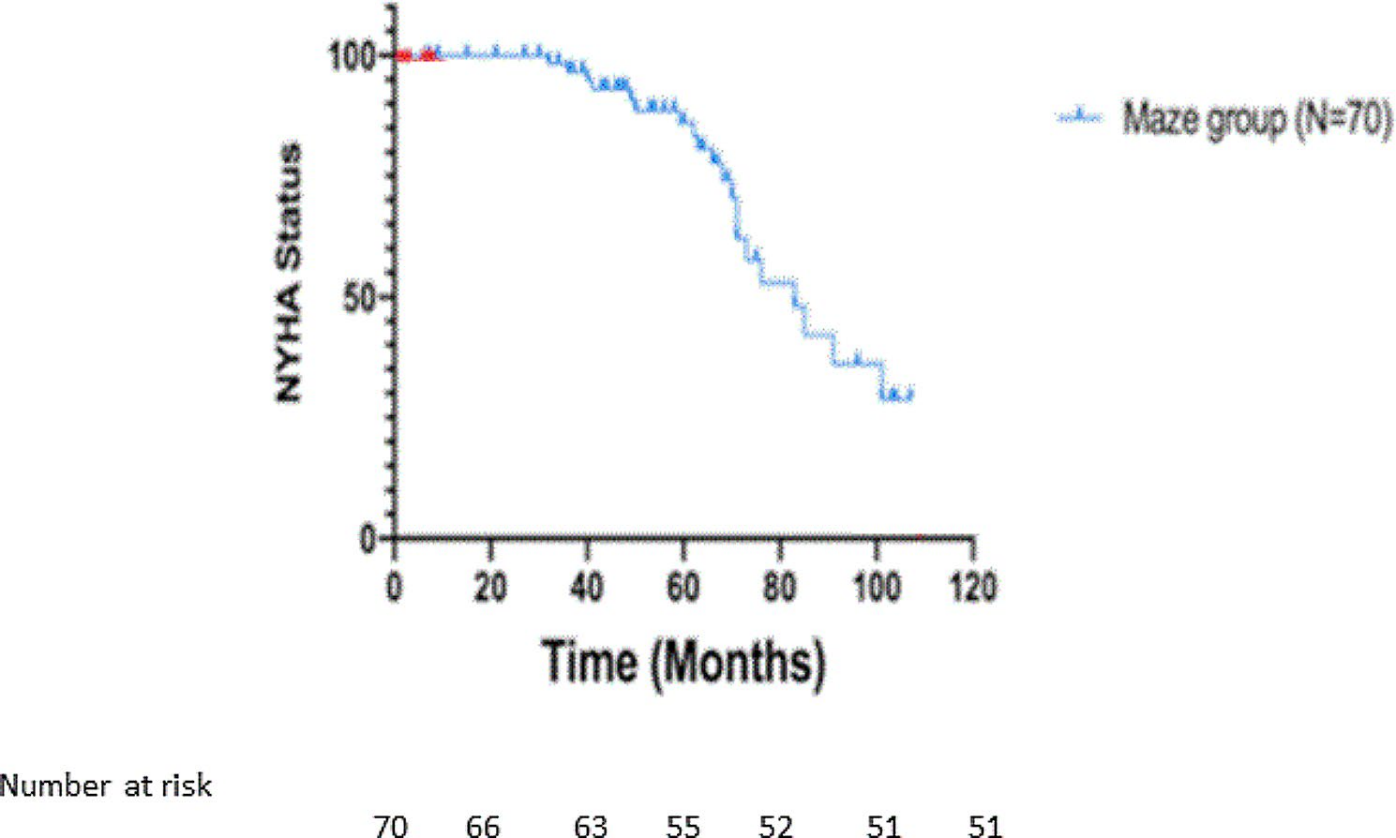
NYHA 1 status in Cox-Maze group

Overall, in the entire cohort, there were 74 patients (31.0%) who remained in NYHA 1 on long-term follow-up. There were clear functional benefits in performing Cox-Maze procedure as shown in Fig. 4. There were 58.8% patients in NYHA 1 status at long-tern follow-up in the Cox-Maze group. On follow-up echocardiography, the LV function did not differ between the study groups.

There were 11 cases of stroke in the overall cohort on long-term follow-up (4.6%). There were 2 patients in the Cox-Maze group (2.9%) compared to 9 patients in the No Surgical AF treatment group (5.3%, *p* = 0.8). Pacemaker rates at long-term follow-up were 7.1% and 8.3% (*p* = 0.33). Only 15 patients in the entire cohort (6.3%) stopped taking oral anticoagulation at long-term follow-up with no comparable difference between the groups (*p* = 0.06).

## Discussion

The Cox-Maze IV has shown excellent success rates with low morbidity and mortality rates and is the only surgical procedure to receive an indication from the Food and Drug Administration for the treatment of AF [16, 17]. However the uptake of concomitant AF ablation has not been universal. This study confirmed that surgical ablation was highly effective in the treatment of AF with 80.0% success at long-term follow-up, consistent with previous studies in elderly patients. Macgregor et al. showed the freedom from atrial tachyarrhythmia on or off anti-arrhythmic drugs was 80% and 61% at 1 and 5 year follow-up respectively in elderly cohort aged > 75 years who had had Cox Maze IV [17]. In another study, Ad and Colleagues showed freedom from atrial tachyarrhythmia after Cox-Maze IV in patients > 75 years was 90%, 85% and 60% at 6 months, 1 and 2 years respectively [18]. Our results were also favorable compared to catheter ablation studies in elderly patients. Bunch et al. showed that 46 patients aged > 80 years reported freedom from AF on or off anti-arrhythmic drugs of 75% and under 30% at 1 and 5 year follow-up after catheter ablation [19].

Importantly, our study clearly demonstrates that surgical ablation did not add perioperative risk even in elderly patients undergoing 2 or more procedures. One of the reasons for the current under-utilisation of surgical ablation is the perception that a concomitant procedure will increase the complexity of the procedure and lead to higher peri-operative risk. Our data refute that argument. Moreover, elderly patients in our study did not experience an increase in renal failure requiring dialysis, reoperation for bleeding, respiratory complications or longer hospital stay. These findings are similar to those previously published by Ad et al. [18], as well as complication rates documented in other studies examining catheter-based ablation of AF in elderly patients [19]. Based on their findings, Ad et al., advocated that age should not be the only discriminatory factor in deciding whether to perform a concurrent Cox Maze procedure [18].

There were 3 patients requiring a PPM post-operatively after Cox Maze procedure (4.3%) which is comparable to the other group in our study. These rates are acceptable as elderly patients experience a greater rate of post-operative PPM compared with younger patients [17], probably from age-related deterioration in sinus node function.

Our study has shown good long-term survival after concomitant Cox-maze IV. The inherent selection bias prevents comparison between our groups as a whole and the numbers propensity matched are too small to compare over the long term. Nonetheless, the sustained maintenance of SR following ablation may confer survival as well as functional benefits. This is clearly demonstrated in previous studies that have shown patients who have surgery without concomitant AF ablation have poorer short and long-term outcomes than patients that come to surgery and are in SR [20, 21]. In addition, AF was found to be an independent significant predictor of long-term mortality [22]. Ngaage et al. demonstrated that pre-operative AF in patients undergoing cardiac surgery was associated with increased morbidity and decreased survival if not corrected [23–25].

There is recent evidence suggesting that anticoagulation can be safely minimized 3–6 months after successful Cox-Maze procedure without increasing the risk of stroke or associated mortality [26], and this would be another advantage of successful ablation. We are pleased with the finding of excellent symptom relief following the Cox-Maze procedure. The assessment of symptoms and quality of life is challenging, especially when part of the symptomatic benefit can be related to the functional improvement as a result of their main cardiac procedure. However, several studies have shown that the return and maintenance of SR for patients with pre-operative AF conveyed a significant increase in quality of life [18, 27, 28]. Ad et al. also demonstrated improved quality of life through SF-12 and AF-specific questionnaire in the elderly cohort > 75 years who had concomitant Cox-Maze IV [18]. Gu et al. showed patients who were restored to SR post-operatively had significantly better NYHA status compared to those in AF [29].

### Limitations

This study is a retrospective and non-randomized study. This means there is interval censoring as well as selection bias of the Cox-Maze group leading to better symptomatic and prognostic benefits in this selected group. Another potential limitation is that the cause of death was not available for all patients. Knowing if the cause of death was cardiac in origin would be of interest as many of these elderly patients carry several comorbid diagnoses as highlighted by the very high Euroscore in the study cohort. Finally, incomplete follow-up for some of the patients may lead to the study suffering attrition and cause reporting biases.

## Conclusions

The outcome of this study suggests that the concomitant Cox Maze procedure in patients > 70 years undergoing multiple cardiac procedures is an excellent procedure for sinus rhythm conversion without increased surgical risks. Our study also demonstrates a significant symptomatic and prognostic benefit of surgical ablation in elderly patients. Ultimately, we feel that age and complexity of surgery should not be contraindications to performing the Cox-Maze procedure.

## Data Availability

Nil required.

## Declarations

### Permission to produce material from other sources

Not required.

### Meeting presentation

1. Oral Presentation at European Association Of Cardiothoracic Surgery (EACTS). Virtual Meeting. Oct 8–10. 2020.

2. AATS Surgical Treatment For Arrhythmias and rhythm disorders. A Virtual Learning Experience. Virtual Poster. (Oct 30–31 2020).
